# Geschichtliche Entwicklung der rekonstruktiven Chirurgie in der Onkologie des HNO-Bereichs

**DOI:** 10.1007/s00106-022-01151-3

**Published:** 2022-03-04

**Authors:** Friedrich Bootz

**Affiliations:** Univ.-HNO-Klinik, Venusberg Campus 1, 53127 Bonn, Deutschland

**Keywords:** Kopf-Hals-Karzinome, Rekonstruktive Maßnahmen, Mikrovaskuläre Lappen, Regionale Lappen, Geschichte, Head and neck cancer, Reconstructive surgical procedures, Microvascular free flaps, Regional flaps, History

## Abstract

Die rekonstruktive Chirurgie ist ein wichtiger Bestandteil der Tumorchirurgie des Kopf-Hals-Bereichs. Große ablative Tumorresektionen wurden bereits Anfang des 20. Jahrhunderts durchgeführt, nachdem es möglich wurde, durch verbesserte Operationstechnik den intraoperativen Blutverlust zu reduzieren bzw. durch Transfusionen auszugleichen. Ein weiterer Meilenstein war die postoperative Infektprophylaxe durch die Einführung der Antibiotika. John Conley erkannte als einer der Pioniere der radikalen Tumorchirurgie die dringende Notwendigkeit rekonstruktiver Maßnahmen. Die Anfänge waren jedoch begleitet von funktionellen postoperativen Einschränkungen, die schließlich erst durch die Einführung des Deltopektoral- und des Pektoralis-major-Lappens verbessert werden konnten. Ein weiterer Schritt war die Einführung der mikrovaskulären Transplantate, die eine bessere, der Situation angepasste Rekonstruktion erlaubte. Anfangs waren jedoch die Komplikationsraten aufgrund mangelnder Technik der Anastomosierung kleiner Gefäße und unzureichender Instrumente sehr hoch. Daher konnte sich diese Methode nur schleppend durchsetzen. Die Techniken der Lappenentnahme und der Mikrogefäßanastomose entwickelten sich jedoch weiter, sodass die mikrovaskuläre Gewebetransplantation zu einer zuverlässigen Methode wurde, die heute zum Standardrepertoire der rekonstruktiven Chirurgie gehört.

## Abstract (en)

Reconstructive surgery is an important part of tumor surgery to the head and neck region. Large ablative tumor resections were already performed at the beginning of the 20th century, after it became possible to reduce intraoperative blood loss through improved surgical techniques or to compensate for it with transfusions. Another milestone was postoperative infection prophylaxis through the introduction of antibiotics. As one of the pioneers of radical tumor surgery, John Conley recognized the urgent need for reconstructive procedures. However, the beginnings were accompanied by postoperative functional impairments, which could only be improved with introduction of the deltopectoral and pectoralis major flaps. Another step was the introduction of microvascular grafts, which enabled better, situation-adapted reconstruction. Initially, however, the complication rates were rather high due to the inadequate technique of anastomosing small vessels as well as inadequate instruments. As a result, these methods were slow to gain acceptance. However, flap harvesting and microvascular anastomosis techniques continued to evolve, making microvascular tissue transplantation a reliable method that is now part of the standard repertoire of reconstructive surgery.

Die Entwicklung der radikalen Tumorchirurgie, die von den Allgemeinchirurgen William Halsted konzipiert und von Hayes Martin umgesetzt wurde, war nur durch Fortschritte bei der Minimierung bis dahin häufig aufgetretener Komplikationen wie Infektionen und Blutverlust möglich. Durch die Verbesserung der Operationstechniken und der Weiterentwicklung des Instrumentariums konnten die Komplikationen reduziert werden. Zusätzlich spielte die Entdeckung der Antibiotika in den 1930er- und 1940er-Jahren eine große Rolle. Dennoch waren komplexere Tumorresektionen durch das Fehlen entsprechender rekonstruktiver Maßnahmen eingeschränkt bzw. hatten erhebliche funktionelle Beeinträchtigungen zur Folge.

Auf dem Gebiet der ablativen Chirurgie von Kopf- und Halstumoren machte sich der HNO-Arzt John Conley aus New York bald einen Namen und erlangte internationalen Ruf. Er war ein früher Verfechter der rekonstruktiven Chirurgie. Bereits 1953 veröffentlichte er einen Artikel dazu [1]. Er propagierte ein einzeitiges Vorgehen der radikalen Tumorexzision mit sofortiger Rekonstruktion, wobei er zur Pharynxrekonstruktion Vollhauttransplantate einsetzte, die jedoch zu erheblicher Vernarbung und Stenosierung führten. Anfangs verwandte er ausschließlich lokale Hautlappen, wie z. B. den Stirnlappen, zur Rekonstruktion in der Mundhöhle bzw. im Oropharynx, die allerdings erhebliche Entstellungen zur Folge hatten. Über ein Jahrzehnt lang war eine Rekonstruktion nur mit denen von Conley beschriebenen lokalen Lappen und Hauttransplantaten möglich, bis der Deltopektorallappen von Bakamjian 1968 entwickelt und bekannt gemacht wurde [2, 3]. In seiner Publikation beschrieb er die Rekonstruktion des Pharynx mit einem medial basierten Deltopektorallappen. Aber auch der Deltopektorallappen hatte häufig erhebliche ästhetische Nachteile zur Folge und verlangte oft ein zweizeitiges Vorgehen, insbesondere bei der Rekonstruktion des Pharynx.

Ein weiterer Schritt in der rekonstruktiven Chirurgie war die Beschreibung des Pektoralis-major-Myokutanlappens durch Ariyan [4]. Er stellte in seiner Publikation die Lappenanatomie und die entsprechende Entnahmetechnik dar, die er erfolgreich bei vier Patienten zum Verschluss großer Defekte im Kopf-Hals-Bereich einsetzte. Diese Technik führte zu einer neuen Ära der Kopf- und Halsrekonstruktion, die es ermöglichte, viele komplexe Gewebsdefekte einzeitig mit einem Lappen mit guten funktionellen Ergebnissen durch den Kopf-Hals-Chirurgen zu verschließen.

Mikrovaskuläre Techniken zur Übertragung von freiem Gewebe entwickelten sich aus der Gefäßchirurgie, wobei mehrere technische Probleme wie das Fehlen einer adäquaten Vergrößerung und die Prophylaxe von Thrombosen ihre frühe Verbreitung verhinderten.

Wesentlich für die Entwicklung der Mikrovaskularchirurgie war neben der Lupenbrille die Einführung des Operationsmikroskops, das in den 1950er-Jahren von Wullstein zusammen mit der Fa. Zeiss, Oberkochen, Deutschland als OPMI 1 entwickelt wurde.

Ein Bericht von Jacobson und Suarez [5] aus dem Jahr 1962 über die Langzeitdurchgängigkeit mikrovaskulärer Anastomosen bei Hunden und Kaninchen leitete eine neue Ära der mikrovaskulären Forschung ein. Bereits 1950 hatte Bernard Seidenberg, ein Gefäßchirurg, 300 Fälle mikrovaskulärer Dünndarmtransplantationen bei Hunden vorgenommen und deren Ergebnisse veröffentlicht [6], bevor er die erste Autotransplantation bei einem Menschen vornahm, der leider den Eingriff nicht überlebte. Im Jahr 1961 beschrieben Roberts und Douglass dann den ersten erfolgreichen Ersatz des zervikalen Ösophagus und des Hypopharynx beim Menschen mithilfe eines revaskularisierten freien Jejunumsegments [7].

Die ersten mikrovaskulären Transplantate in der Kopf-Hals-Chirurgie wurden dann in den 1970er-Jahren an mehreren Institutionen klinisch eingesetzt, wobei der Erfolg lediglich bei etwa 70 % lag. William Panje und Shan Baker veröffentlichten 1977 die erste Serie von Rekonstruktionen der Mundhöhle mit einem freien Leistenlappen [8]. Der mikrovaskuläre Gewebetransfer konnte sich jedoch wegen Problemen bei der Anastomosierung der kleinen Gefäße mit sehr häufig auftretenden Gefäßverschlüssen und dadurch bedingten erheblichen postoperativen Komplikationen nicht durchsetzen. Auf der Suche nach anderen rekonstruktiven Techniken kam man wieder auf die gestielten, insbesondere den Pektoralis-major-Lappen zurück, die sich als sehr zuverlässig erwiesen und die weitere Entwicklung des mikrovaskulären Gewebetransfers behinderten. Bald entwickelten sich der Pektoralis-major- und der Deltopektorallappen zu den Standardinstrumenten der rekonstruktiven Chirurgie des Kopf-Hals-Bereichs. Auch Conley sprach sich für die Überlegenheit der gestielten Lappen gegenüber dem freien Gewebetransfer aus und hat diese noch in der zweiten Auflage seines Standardwerks, das im Jahr 1989 erschien, ausführlich dargestellt.

Man erkannte jedoch bald die Grenzen der gestielten Transplantate. Nicht alle Regionen des Kopf-Hals-Bereiches konnten damit erreicht werden, und der Gefäß-Muskel-Stiel war oft ein Hindernis. Häufig war ein zweizeitiges Vorgehen notwendig. Ferner waren die funktionellen und ästhetischen Ergebnisse meist nicht zufriedenstellend.

Die besonderen Erfordernisse der Rekonstruktion nach ausgedehnten Tumorresektionen und die höheren Ansprüche an die funktionellen und ästhetischen Ergebnisse sorgten dafür, dass die Techniken des freien Gewebetransfers wieder aufgegriffen und weiterentwickelt wurden.

Die exakten anatomischen Studien durch McGregor im Jahr 1973 über Gefäßverläufe, Gefäßdurchmesser und Versorgungsgebiete der zuführenden Gefäße verschiedener Körperregionen und der Definition der „axial pattern flaps“ stellten die Basis zur weiteren Entwicklung unterschiedlicher Transplantate dar [9]. Der revaskularisierte Latissimus-dorsi-Lappen wurde bereits Ende der 1970er-Jahre von Olivari beschrieben [10, 11]. 1979 beschrieb Taylor den revaskularisierten Beckenkamm an der A. circumflexa ileum profunda [12], der sich zu einem Standardtransplantat zur Rekonstruktion des Unterkiefers entwickelte und heute noch in dieser Form eingesetzt wird. Die Fibula war jedoch der erste Knochen, der mit Erfolg als vaskularisiertes Transplantat eingesetzt wurde, und zwar zur Überbrückung ausgedehnter Langknochendefekte, wie von Taylor bereits 1975 berichtet [13]. Zur Rekonstruktion des Unterkiefers wurde dieses Transplantat allerdings zum ersten Mal 1989 von Hidalgo eingesetzt [14]. Heute stellt die Fibula das Standardtransplantat zur Rekonstruktion des Unterkiefers dar.

Die Bedeutung des Rektus-abdominis-Lappens, der sich v. a. in den USA zum „Arbeitspferd“ der rekonstruktiven Chirurgie entwickelte, wurde von Brown et al. bereits 1975 erkannt [15]. 1980 wurde von Dos Santos der Skapulalappen [16] und 1982 von Nassif der Paraskapulalappen [17] beschrieben, beide an der A. circumflexa scapulae.

1978 berichteten Yang et al. [18] über den fasziokutanen Unterarmlappen, der 1982 von Mühlbauer et al. [19] außerhalb Chinas bekannt gemacht wurde. Er fand jedoch anfangs durch die hohen Komplikationsraten an der Entnahmeregion und durch den hauptsächlich von Kieferchirurgen vorgenommenen Einsatz des antimesenterisch aufgeschnittenen Jejunumsegments als Patch keine weite Verbreitung bei der Rekonstruktion in der Mundhöhle und im Pharynx. Die ersten klinischen Anwendungen des Jejunumpatchs wurden im Jahr 1971 von Black, Bevin und Arnold vorgenommen [20]. Die Technik geht auf Green und Som zurück, die erstmals 1966 solche Transplantationsversuche an Hunden durchführten [21]. Das Jejunumpatch wurde aufgrund seiner Schleimhautähnlichkeit und der ausgesprochen guten Modellierbarkeit favorisiert und anderen Transplantaten vorgezogen.

Soutar berichtete im Jahr 1986 über eine große Zahl von Rekonstruktionen nach Tumorresektion im Oropharynx und in der Mundhöhle mithilfe des Unterarmlappens [22]. In England hatte sich diese Methode an einigen wenigen Zentren zur Standardrekonstruktion entwickelt. Der Autor hatte bereits 1987 bei Philip Stell in Liverpool während eines einjährigen Aufenthalts die Gelegenheit, diese Technik zu erlernen und sie danach in Deutschland im Kopf-Hals-Bereich einzuführen. Allerdings war es anfangs aufgrund der als sehr hoch angesehenen Entnahmemorbidität [23, 24] schwierig, die Kopf-Hals-Chirurgen, insbesondere die Kieferchirurgen, von der Qualität des Transplantats zu überzeugen. Die klinische Erfahrung hat jedoch gezeigt, dass das Jejunumpatch mechanisch wenig belastbar ist und zu nicht unerheblicher Schrumpfung insbesondere nach Bestrahlung neigt. Häufig traten v. a. in mechanisch belasteten Regionen der Mundhöhle Ulzerationen, Blutungen und Nekrosen auf. Diese negative Eigenschaft des Jejunumtransplantats führte zu einem Aufschwung der fasziokutanen Lappen, insbesondere des Unterarmlappens, der mechanisch gut belastbar ist, nur eine geringe Schrumpfungsneigung zeigt und fast ebenso gut modellierbar ist wie das Jejunumsegment, allerdings keine schleimhautähnliche Eigenschaft besitzt.

Durch eine verbesserte Operationstechnik bei der Entnahme des Unterarmlappens konnte zudem die Morbidität deutlich gesenkt werden [25]. 1991 konnten wir unsere Erfahrungen in einem Buch [26] veröffentlichen und als Koautor einen MKG-Chirurgen gewinnen. Von dem Zeitpunkt an war das Unterarmtransplantat auch von MKG-Chirurgen akzeptiert und löste im Laufe der Zeit das Jejunumpatch zur Rekonstruktion im Bereich der Mundhöhle und des Pharynx ab.

Zur Rekonstruktion des Hypopharynx nach Pharyngolaryngektomie wurde über längere Zeit hinweg noch das Jejunum als Rohr eingesetzt. Doch eine nicht unerhebliche Anzahl von Komplikationen sowohl in der Empfängerregion als auch an der Entnahmestelle hat auch in diesem Bereich dazu geführt, dass nach anderen Methoden gesucht wurde. Harii beschrieb 1985 als Erster die Rekonstruktion des Hypopharynx mithilfe eines zum Rohr geformten Unterarmlappens [27]. Unsere Arbeitsgruppe hat daran eine Modifikation vorgenommen. Der Unterarmlappen wird nicht mehr zum Rohr geformt, sondern U‑förmig an die prävertebrale Faszie angeheftet [28].

Die Mikrogefäßanastomose hat seit ihrer Erstbeschreibung durch Carell, abgesehen von der Verbesserung des Nahtmaterials, des Instrumentariums, der Gefäßclips und der Vergrößerungsmöglichkeiten, keine wesentliche Entwicklung durchgemacht. Bereits Seidenberg verwandte 1958 eine Art von Konnektorsystem. Konnektoren wurden zwar weiterentwickelt, konnten jedoch die Mikrogefäßanastomose mit Nahttechnik bis heute nicht vollständig ersetzen.

Die Entwicklung des mikrovaskulären Gewebetransfers hat zu einer enormen Bereicherung der rekonstruktiven Chirurgie nach Tumorresektion im Kopf-Hals-Bereich beigetragen und gehört heute zum Standardrepertoire der Kopf-Hals-Onkologie. Es gibt eine hohe, fast unüberschaubare Vielfalt an freien Transplantaten, die nicht alle für die rekonstruktive Chirurgie des Kopf-Hals-Bereichs geeignet erscheinen. Der verantwortungsbewusste Operateur wird sich ein Repertoire an mikrovaskulären Lappen zulegen, mit dem es ihm möglich ist, den rekonstruktiven Anforderungen seines Fachgebiets gerecht zu werden. Neben mikrovaskulären Transplantaten, die aufgrund ihrer hohen Flexibilität sicherlich die erste Wahl sind, gibt es auch Situationen, in denen heute noch gestielte Lappen eingesetzt werden müssen, sodass sie nicht aus dem Repertoire der rekonstruktiven Techniken gestrichen werden dürfen.

Prälaminierten Lappen, bei denen z. B. auf Muskel- oder entepithelisierten fasziokutanen Lappen spezifisches Gewebe aufgebracht wird, können ebenfalls zur situationsgerechten Rekonstruktion eingesetzt werden. Solche Techniken werden seit mehreren Jahren beschrieben, wobei oft Schleimhaut aufgebracht wird [29, 30]. Zukünftige Entwicklungen können in Verfahren liegen, bei denen durch „tissue engineering“ bedarfsgerechte Transplantate mit z. B. Schleimhaut, Knochen oder Knorpel geschaffen werden, wobei jedoch die bisherigen Ergebnisse eher ernüchternd sind. Ein Nachteil dieser Techniken ist darin zu sehen, dass eine einzeitige Rekonstruktion nach Tumorresektion nicht möglich ist. Diese neuen Ansätze zeigen die vielfältigen weiteren Möglichkeiten mikrovaskulärer Transplantate in der rekonstruktiven Chirurgie der Onkologie des Kopf-Hals-Bereichs.
